# Rabies vaccinations at the rural–urban divide: successes and barriers to dog rabies vaccination programs from a rural and urban campaign in Zambia

**DOI:** 10.3389/fvets.2024.1492418

**Published:** 2025-01-20

**Authors:** Ricky Chazya, Chilufya Aneta Susan Mulenga, Andrew D. Gibson, Frederic Lohr, Cassandra Boutelle, Sarah Bonaparte, Oline Sinywibulula, Gareth Thomas, Patricia Bwalya, George Dautu, Linous Munsimbwe, Geoffrey Muuka, Luke Gamble, Ryan M. Wallace, Michelle A. Waltenburg

**Affiliations:** ^1^Ministry of Fisheries and Livestock, Lusaka, Zambia; ^2^Mission Rabies, Cranborne, Dorset, United Kingdom; ^3^National Center for Emerging and Zoonotic Infecious Diseases, U.S. Centers for Disease Control and Prevention, Atlanta, GA, United States

**Keywords:** rabies, rabies vaccination, dog vaccination programs, dog-mediated rabies, dog-mediated human rabies elimination

## Abstract

**Introduction:**

Dog vaccination against rabies is considered one of the most effective strategies at preventing human deaths from rabies and is a key strategy for eliminating dog-mediated human rabies deaths. Traditional vaccination approaches in Zambia rarely collect operational data to assess coverage and inform subsequent campaigns.

**Methods:**

Following mass vaccination campaigns in rural (Itezhi tezhi) and urban (Lusaka) communities, we evaluated vaccination coverage achieved during the campaigns and characterized and estimated the dog population in these communities.

**Results:**

Herd immunity (i.e., 70% vaccination coverage) was not achieved in the Lusaka campaign, likely due to challenges in pre-campaign community sensitization and distance to vaccination sites in the central point campaign approach. Dog population density showed a strong exponential association with human density (R^2^ = 0.89). Extrapolating this relationship nationally, there are an estimated 3.2 million dogs in Zambia (human-to-dog ratio 5.8:1) with 86% residing in rural communities at a very low density of less than 6 dogs per square kilometer.

**Discussion:**

As most dogs were found to reside at very low densities, unique challenges to large-scale dog vaccination approaches may impact Zambia, due to high logistical costs associated with these settings. Prioritizing vaccinations in higher-density free-roaming dog populations could maximize effectiveness in resource-limited settings. Private veterinary services were commonly utilized among surveyed dog owners in urbanized communities in Lusaka, suggesting that they are an important collaborator for achieving rabies herd immunity. With improved knowledge of dog population and ownership characteristics, Zambia is well-prepared to design more effective vaccination campaigns as the rabies elimination program expands.

## 1 Introduction

Rabies is one of the most lethal infectious diseases, with the highest case fatality rate of all zoonoses. Bites from dogs are responsible for approximately 95% of the estimated 70,000 human rabies deaths globally (1). Over 70% of the world’s population resides in areas where dogs are a reservoir for rabies (2). Asia and Africa have the highest risk for rabies transmission, attributed to relatively low herd immunity in dog populations and propensity for dogs to roam freely within the community.

Rabies is an endemic disease in Zambia, with an average of 20 human rabies deaths recorded annually, but likely many more that go undocumented (3). While rabies is a notifiable disease in Zambia, laboratory confirmed cases in humans have been rare due to low rates of community and healthcare reporting, lack of capacity for invasive autopsies, and cultural hesitancy for post-mortem examinations. The reported number of people bitten by dogs and who require post-exposure prophylaxis is increasing (4), thus, rabies has been ranked as one of the top five priority zoonotic diseases in Zambia. In 2023, Zambia’s dog rabies control strategy was officially certified by the World Organization for Animal Health and is a major step towards the goal of eliminating dog-mediated human rabies in the country by 2030 (3).

Most rabies cases in animals and humans are caused by the dog-maintained rabies virus variant, which is mainly transmitted by domestic, free-roaming dogs (2). Dog vaccination against rabies is considered one of the most effective strategies at preventing human deaths from rabies and is key for rabies elimination. Observations on the relationship between rabies incidence and vaccination coverage in dogs have shown that 70% coverage is necessary to eliminate or prevent rabies outbreaks (5, 6). This critical percentage is not always achieved in mass vaccination campaigns, particularly in communities with high densities of free-roaming dogs and among dog populations that do not typically receive routine veterinary services (7). Most vaccination campaigns in Zambia have thus far relied on central point (CP) vaccination, which typically involves the strategic placement of government dog vaccination teams throughout a community with the expectation that dog owners will bring their dogs to the campaign (8). Despite the Ministry of Fisheries and Livestock (MFL) in Zambia conducting government-sponsored rabies vaccination campaigns since at least 2013, rabies cases continue to persist in these communities, suggesting that critical vaccination thresholds to halt enzootic transmission are not being met (8, 9). The COVID-19 pandemic, however, has likely exacerbated this situation by disrupting routine vaccination services and limiting both access to and utilization of health interventions, which may have contributed to increased outbreaks of rabies and other infectious diseases (10). While a lack of understanding of the dog population should not delay vaccination programs, persistent rabies cases in the face of dog vaccination programs should raise concern about the vaccination coverages achieved. In communities with persistent rabies cases, collection of operational data to assess coverage and to inform subsequent campaigns is recommended (11–13).

As part of a comprehensive vaccination program, studies to characterize the dog population (including population estimates and ownership characteristics) can be conducted in parallel to vaccination campaigns and lead to more effective vaccination strategies (13–16). However, dog population estimates are not always conducted due to the cost, lack of expertise, and loosely defined methodologies (12). Rabies programs typically describe dog populations in terms of the human to dog ratio (HDR); however, the HDR is not constant across all communities and can differ greatly based on community characteristics, religion, poverty, and other factors (14, 15). Therefore, reliance on a static HDR can lead to inconsistent post-vaccination herd immunity and persistent rabies cases. The MFL in Zambia has historically conducted mass vaccination campaigns in Lusaka using an estimated HDR of 45:1, although this information has not been validated (17).

We conducted post-vaccination evaluations to assess vaccination coverage achieved during two mass vaccination campaigns in urban Lusaka District, Zambia in July 2022 and rural Itezhi tezhi District, Zambia in September 2021 and estimated and characterized the dog population in these areas.

## 2 Materials and methods

### 2.1 Vaccination campaign

#### 2.1.1 Study locations

**Figure 1:**
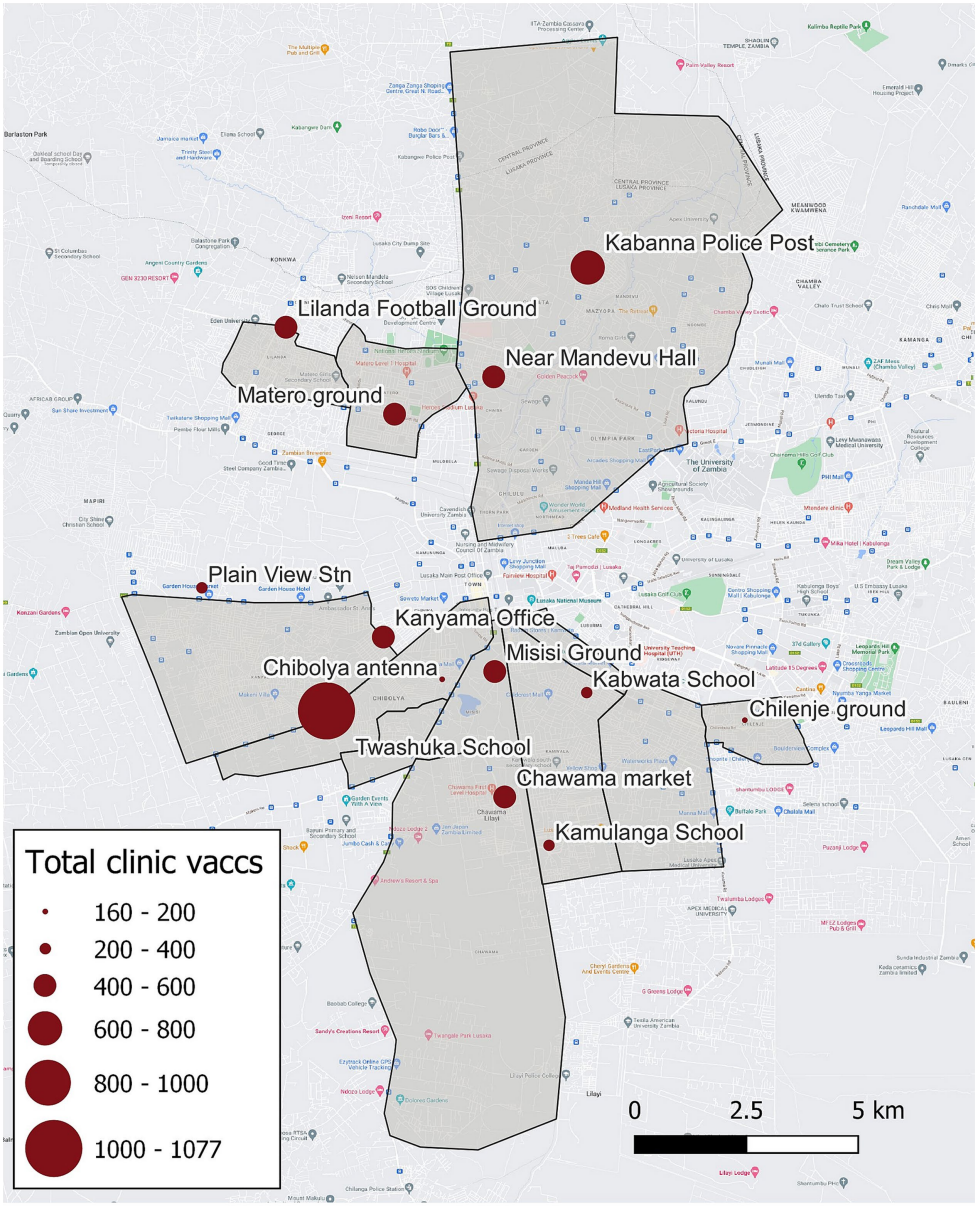
Vaccination zones, central point vaccination locations, and total number of vaccinations administered—Lusaka, Zambia, 2022. Grey polygons indicate regions targeted by the campaign (i.e., vaccination zones), red dots indicate central point vaccination locations, and size of the red dots corresponds to the number of vaccinations administered.

**Figure 2:**
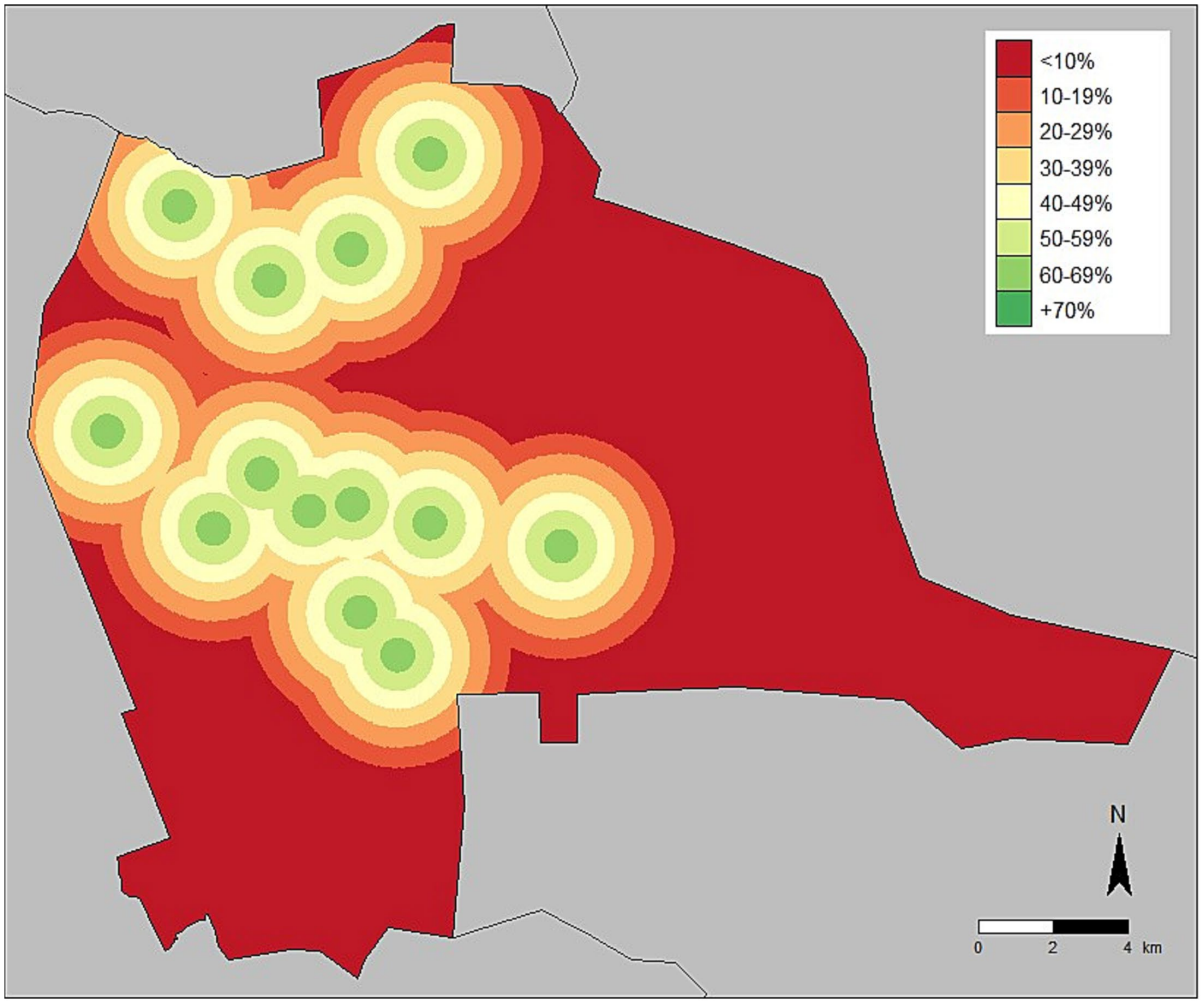
Vaccination coverage adjusted for distance from central point vaccination location—Lusaka, Zambia, 2022.

For the Lusaka campaign, MFL identified 13 CP vaccination location sites (hereafter referred to as CP sites) within nine urban and peri-urban administrative areas in Lusaka District (Figure 1, 2). Regions vaccinated by the Lusaka campaign (i.e., vaccination zones) varied greatly in terms of human density and urbanization; most were highly urban with no open fields or industrialized areas (Supplementary Table 1). Lusaka is the capital of Zambia, with an average human population density among the vaccination zones of 11,330 people per square kilometer (range: 8,600–13,400).

**Figure 3:**
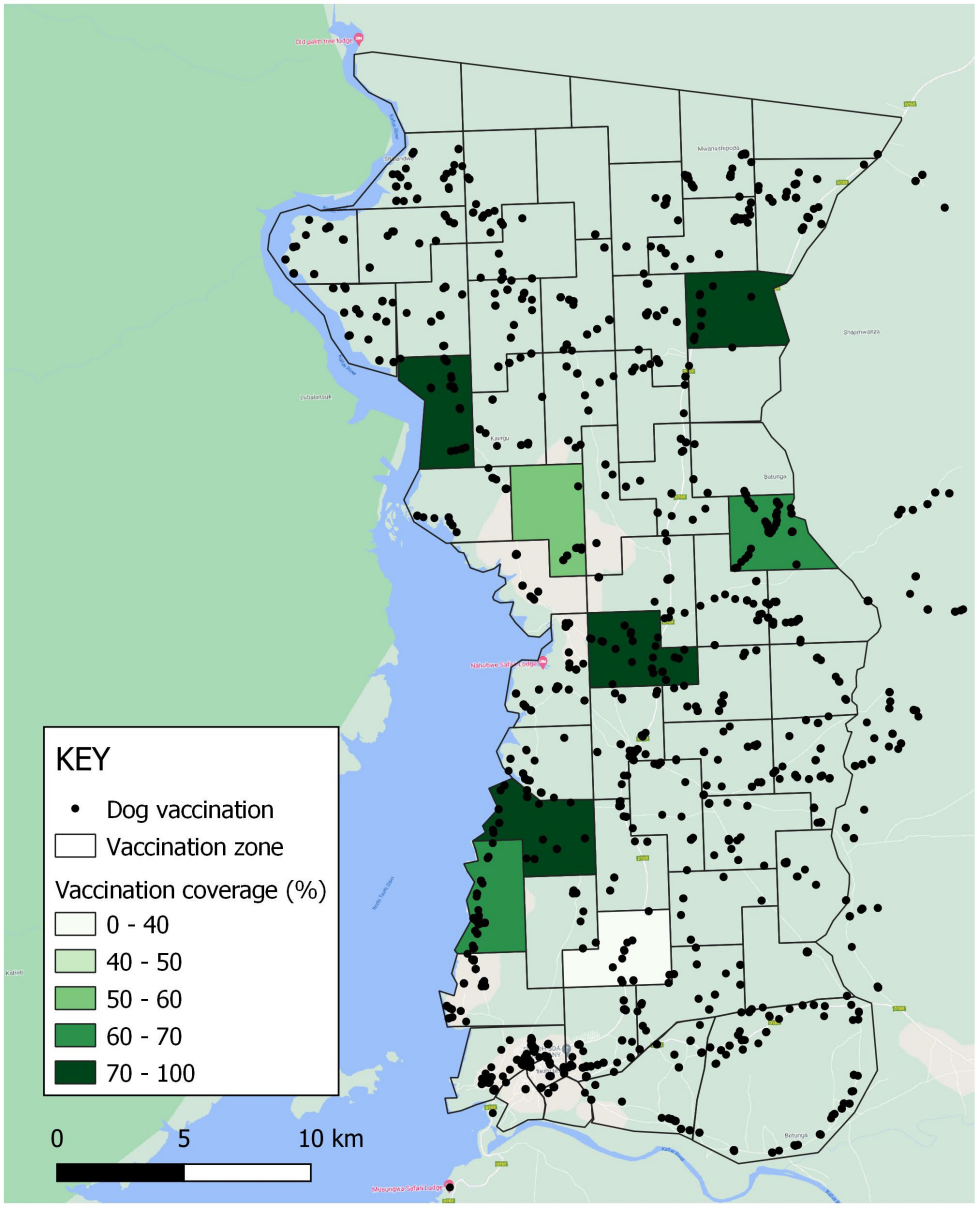
Vaccination zones, vaccinations administered, and estimated vaccination coverage in Itezhi tezhi district, Zambia, 2021. Black dots represent locations where vaccinations were administered.

For the Itezhi tezhi campaign, MFL, Zambia National Public Health Institute (ZNPHI), Game Rangers International (GRI), and the International Rabies Taskforce (IRT) identified several CP sites within seven administrative areas in rural Itezhi tezhi District (Figure 3). Itezhi tezhi is a pastoral community in Southern Zambia with part of its land hosting the Kafue National Park, with an average human population density of 53 people per square kilometer (range: 29–114; Supplementary Table 1).

#### 2.1.2 Campaign planning

Local coordinators selected vaccination zones using historical data from previous campaigns conducted in Zambia over the last 10 years. CP sites were based on knowledge of dog population distributions and ease of community access, directing vaccination teams to community landmarks (e.g., markets, schools, churches, clinics, etc.).

In Lusaka, coordinators assigned four vaccination teams, each comprised of four staff, to each CP site for up to 3 days. Teams remained in locations until vaccination teams, field managers, or campaign coordinators decided that adequate vaccination coverage had been achieved, although no quantitative methods were conducted to inform this decision. Based on past campaign experience and using a HDR of 45:1, a goal of vaccinating approximately 10,000 dogs was set to reach target vaccination coverage of 70% (17).

In Itezhi tezhi, coordinators assigned daily working areas to each vaccination team (three staff total) via a smartphone-web system, the WVS App, to visualize the targeted area (12). The teams began each day assigned to a CP site. After all animals brought to the CP sites were vaccinated, the teams moved door-to-door within the community to cover the entire vaccination zone allocated to them for that day. Teams used the path tracker function on the WVS App to guide them.

#### 2.1.3 Data collection

Vaccination teams used the WVS app to collect and aggregate vaccination data during the campaign (12). Vaccination teams collected GPS location, time, date, and username for every dog vaccinated. Teams collected data offline at the time of vaccination and submitted via cellular internet connection at the end of the day.

In Lusaka, a vaccination team consisted of one veterinary technician in charge of vaccinating, one assistant technician in charge of data entry using the WVS app, another assistant responsible for writing a vaccination certificate, and one dog handler. In Itezhi tezhi, a vaccination team consisted of one veterinarian or veterinary assistant, one data collector, and one assistant. Vaccination teams underwent training on use of the WVS App several days prior to the campaign. Trained technicians administered 1.0 mL dose of Nobivac® Rabies vaccine (MSD Animal Health, United States) subcutaneously. Assistants marked vaccinated dogs with a temporary wax crayon on the forehead for identification in post-vaccination surveys and prevention of repeated vaccination.

Field managers directed vaccination teams, reviewed the area vaccinated each day, managed bite exposures, and ensured that vaccination data was uploaded within the WVS App.

#### 2.1.4 Public awareness and community sensitization

In Lusaka, campaign coordinators engaged the District Commissioner to mobilize their communication networks to publicize the vaccination campaign. Announcements were made during local church services a week before the campaign. While there was no concerted advertising campaign, two radio stations and television stations ran announcements for the campaign. Public awareness for the campaign was heavily reliant on vaccination teams mobilizing their own community networks by making announcements by megaphone in key locations (e.g., markets, schools, churches, etc.) a day before the campaigns. The Lusaka campaign ran from July 4 to 21, 2022.

In Itezhi tezhi, campaign coordinators advertised the vaccination campaign within the targeted communities by speaking to Village Action Groups, schools, churches, and on the radio. Vaccination teams conducted additional sensitization daily using megaphones as they moved within their assigned daily working areas. The Itezhi tezhi campaign ran from September 6 to 17, 2021.

### 2.2 Post-vaccination evaluation

#### 2.2.1 Dog sight surveys

In Lusaka, field survey teams conducted dog sight surveys in six vaccination zones to gather data needed to estimate the free-roaming dog population and assess free-roaming dog vaccination coverage by documenting the presence of a vaccination mark (e.g., wax paint). Teams completed dog sight surveys over two consecutive days to allow for a sight/re-sight analysis using the Lincoln-Peterson formula with data and photographs captured in the WVS App (18–20). These teams were independent from the vaccination teams to ensure that no bias was introduced through knowledge of the areas that had been vaccinated.

#### 2.2.2 Household surveys

Field survey teams conducted household surveys in nine vaccination zones in Lusaka and eight in Itezhi tezhi to estimate vaccination coverage and the owned dog population (free-roaming and confined). Teams administered a standardized questionnaire to consenting adults (≥18 years) which was used to determine vaccination coverage and HDRs (Supplementary Table 2). Teams initiated post-vaccination household surveys in a vaccination zone once the field managers indicated they were complete.

In Lusaka, when owned dogs were missing data on confinement status (i.e., free roaming, partially confined, always confined), we imputed confinement status based on reported confinement status for other dogs in the household or randomly assigned based on the confinement status proportions across all dogs in the survey with known information. We applied within-home imputation to 19% (*n* = 37) of owned dogs. We randomly assigned confinement status for 20% (*n* = 40) of owned dogs. When owned dogs were missing data on recent vaccination (i.e., rabies vaccination received within the last year, excluding the campaign), we imputed vaccination based on reported vaccination for other dogs in the household. We considered dogs with unknown vaccination status reported by the owner not vaccinated. We imputed vaccination for 18% (*n* = 36) of owned dogs. We determined owned-dog vaccination coverage by calculating the total number of dogs reported as vaccinated in the last year by the owner (including during the recent campaign) out of the total dog population reported as owned by survey respondents. Proof of vaccination was not required.

Trained interview teams conducted interviews in English by reading aloud from a script and recording answers on an electronic form in the WVS App. As for dog sight surveys, staff were independent of vaccination teams. Staff obtained informed consent from each person prior to beginning the interview.

#### 2.2.3 Dog ownership characteristics and vaccination coverage

In Lusaka, we asked dog owning households a series of questions to determine the distance they self-reported they were willing to walk their dog to a rabies vaccination center. Respondents were first asked if they would walk 500 meters. A positive response led to a series of similar questions with distances of 1, 1.5, 2, and 3 km. If at any time respondents provided a negative response, the last affirmative response was considered their maximum distance willing to walk. A negative response led to a series of questions with distances of 250 and 100 m. If at any time respondents provided a positive response, this was considered the maximum distance they were willing to walk. Respondents that claimed they would walk more than 3 km or less then 100 m were asked to provide their maximum distance. We also asked dog owners about their perceptions towards dogs and the type of care they provide to their dogs. We scored dog owners’ perceived value of their dogs by summing responses to these survey questions. We awarded one point per question for responses expressing that owners value their dogs, we deducted one point per question for responses expressing that owners did not value their dogs, and we assigned no points for neutral responses (Supplementary Table 2, questions 504–509).

**Figure 4:**
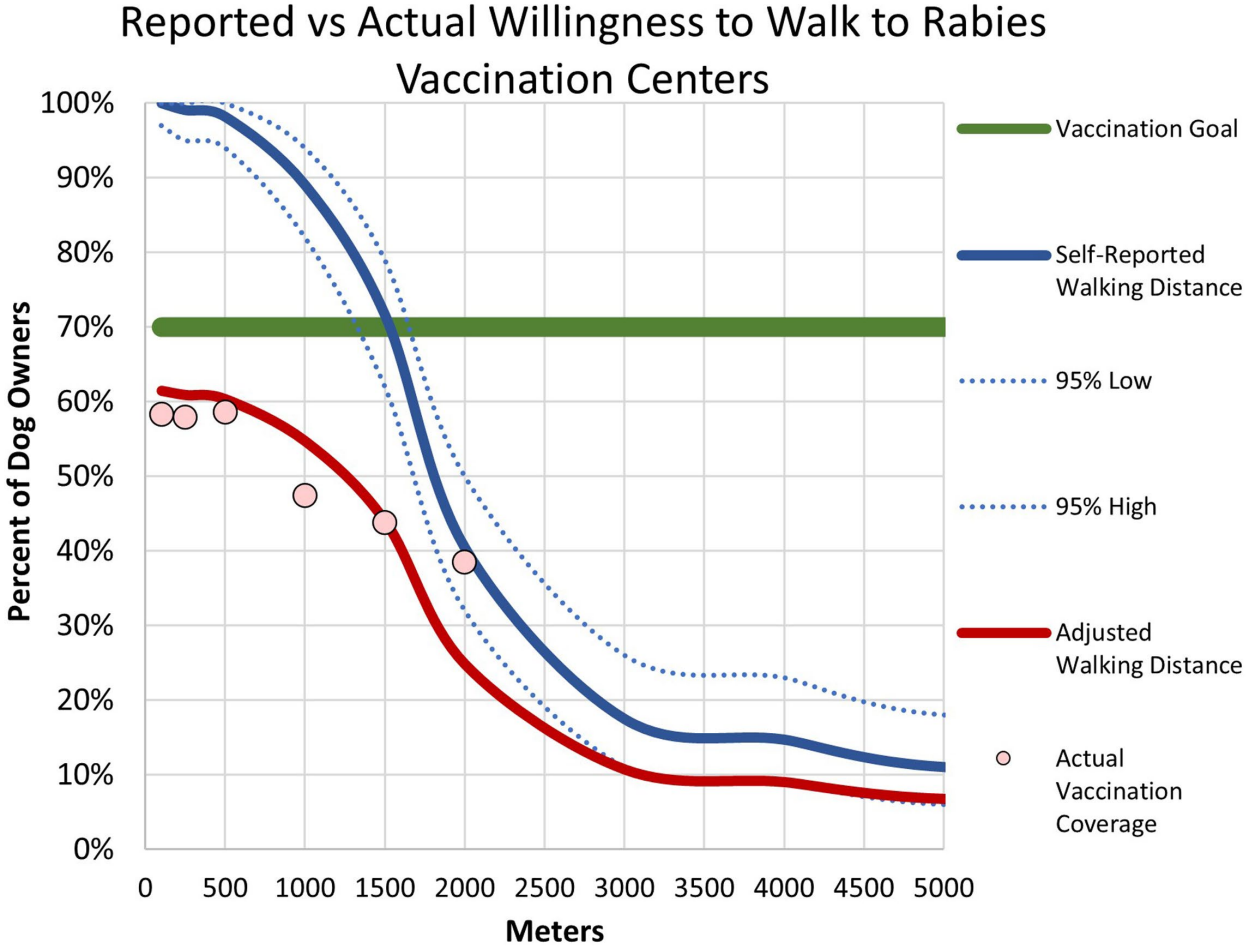
Association between willingness to walk to a central point vaccination location and vaccination coverage–Lusaka, Zambia, 2022. The blue line represents household survey respondents self-reported willingness to walk to a central point vaccination location. The dotted blue lines represent 95% confidence intervals. The red line represents adjusted willingness to walk distances. The pink dots represent household survey-derived vaccination coverage.

In Lusaka, we estimated road distance and walking time between each survey respondent’s home and the nearest CP site using Google Maps.^1^ We calculated vaccination coverages by distance to the nearest CP site through three methods: (a) the overall coverage among all dogs; (b) the coverage among dogs that were vaccinated by a private veterinarian; and (c) the coverage among dogs when only considering those that were not already vaccinated through private means. We down adjusted self-reported willingness to walk to reflect the actual dog vaccination coverage, by distance, (method c), and we considered this the “actual distance willing to walk” (Figure 4).

#### 2.2.4 Dog population estimates

We derived dog population estimates using the method previously published by Wallace and Moran (14). We analyzed data collected during dog sight surveys using the Simple Features package (sf) in R to add a sightline buffer to the dog sight survey path (100 m), calculate the transect length (km), estimate the human population along the path, and apply the Lincoln-Petersen formula for the dog population along the path (19).

We applied a stepwise approach to determine the overall dog population demographics to vaccination zones where both dog sight surveys and household surveys were conducted, only dog sight surveys were conducted, and only household surveys were conducted using previously published methods (14). We overlayed a grid of 0.88 km^2^ hexagons across the country using Uber’s H3 hexagonal hierarchical geospatial indexing system via the H3jsr R library and calculated the human population for each hexagon from Meta’s High Resolution Population Density raster files using the exact_extract() function within the exactextractr R (21). We used the extracted population estimates from all hexagons that intersected the dog sight survey path or were within a set distance (1.5 km for Lusaka, 0.5 km for Itezhi tezhi) of the household survey site to estimate the human population density for each survey site. We tested associations between Meta’s estimated human and survey-derived dog populations by logarithmic, linear, and exponential models, and based the best fit on the highest R^2^ value (14). We calculated the proportion of dogs that are owned-confined, owned-roaming, and unowned for each study location.

**Figure 5:**
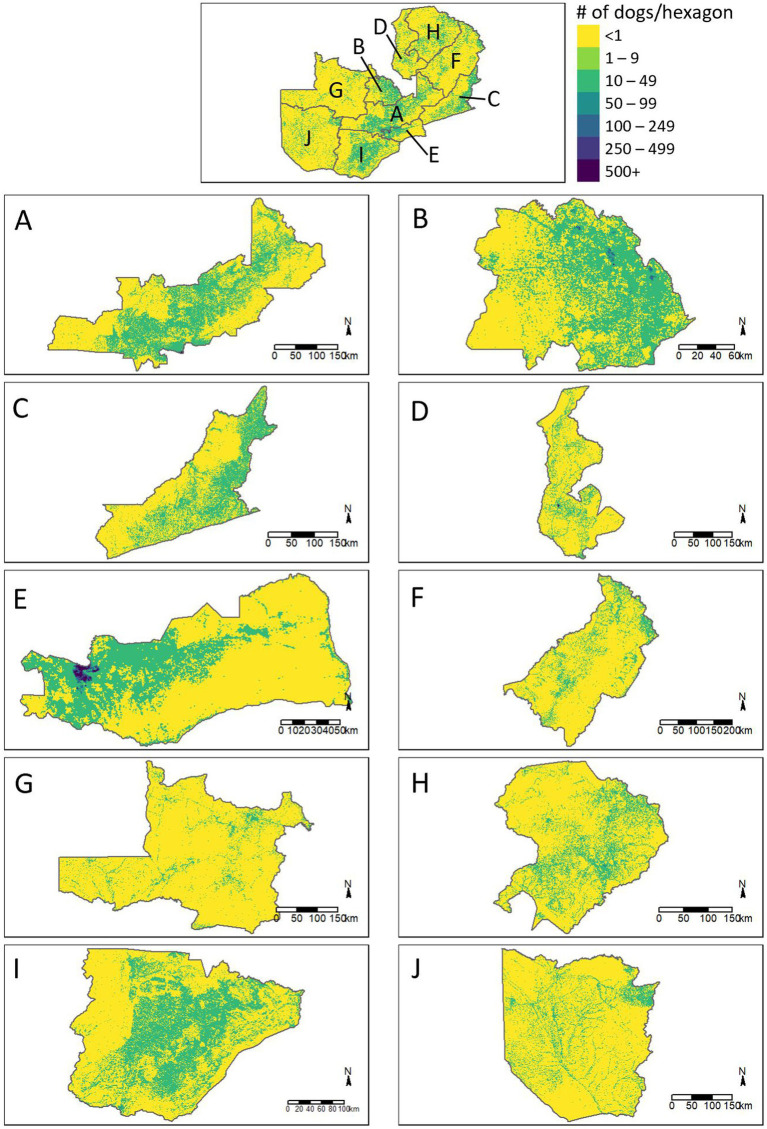
Estimated distribution of dogs across the provinces of Zambia. **(A)** Central Province, **(B)** Copperbelt Province, **(C)** Eastern Province, **(D)** Luapula Province, **(E)** Lusaka Province, **(F)** Muchinga Province, **(G)** North Western Province, **(H)** Northern Province, **(I)** Southern Province, **(J)** Western Province.

We applied the best fitting density functions across the entire country to estimate dog population demographics. We derived the human population for each cell of a country-wide hexagonal grid using the same methods as detailed above, and calculated dog population density in each hexagon using the function of best fit (Figure 5). For further exploration of the dog population density results, we categorized hexagons by human population density (>5,000 people/km^2^, 500–4,999 people/km^2^, 50–499 people/km^2^, 5–49 people/km^2^, 1–4 people/km^2^) and urbanicity level (urban = areas with population density of at least 500 people/km^2^, peri-urban = areas with population density 50–499 people/km^2^, rural = areas with fewer than 50 people/km^2^). We summarized results across Lusaka District and all of Zambia.

## 3 Results

### 3.1 Vaccination campaign

**Figure 6:**
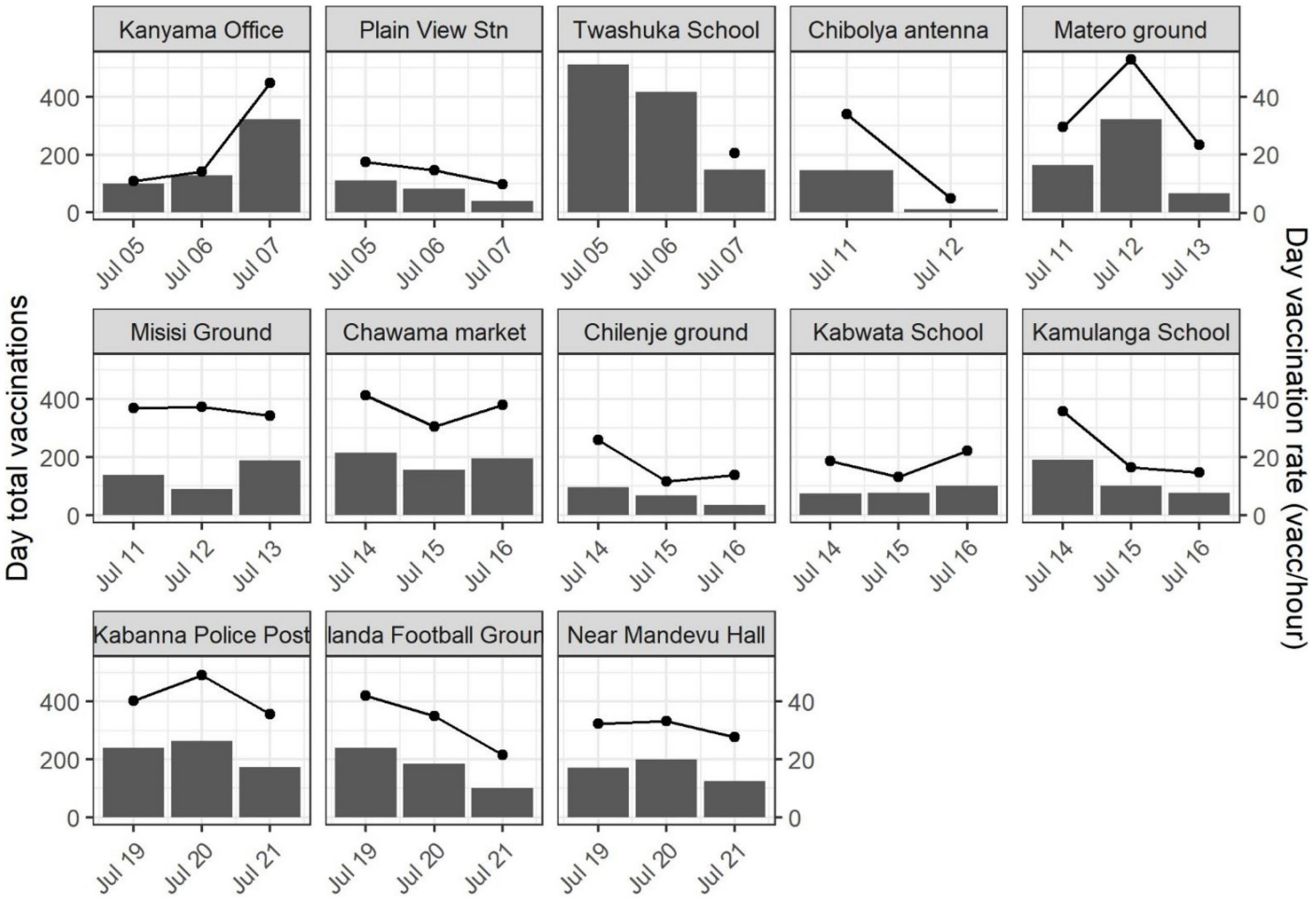
Total daily vaccinations by central point vaccination location (bars, left y-axis) and mean hourly rate of vaccination (lines, right y-axis)—Lusaka, Zambia, 2022.

Field teams vaccinated a total of 6,054 dogs during the Lusaka Campaign between July 4–21, 2022 for an average of 143 dogs vaccinated per team per day (95% confidence interval [CI]: 117–168). The rate of vaccination was typically highest on the first day at a CP site (mean: 156 dogs/team/day), decreasing to the third day (130 dogs/team/day); however, this pattern was not observed at all locations (Figure 6). Field teams vaccinated a total of 3,930 dogs during the Itezhi tezhi campaign between September 6–14, 2021 for an average of 71 dogs vaccinated per team per day (95% CI: 62–79).

### 3.2 Post-vaccination evaluation

#### 3.2.1 Dog sight surveys

Field teams conducted dog sight surveys in six vaccination zones over 4 days during the Lusaka campaign. Overall, staff recorded 143 free-roaming dogs on day 1 and 113 on day 2. On day 2, the average day 1 detectability of the dog population was 19.6% (survey site range: 7–59%). Of the 191 unique free-roaming dogs observed during the dog sight surveys, 56 (29%) had evidence of vaccination (i.e., paint mark). Field teams covered a total of 46 linear kilometers in dog sight surveys, resulting in four dogs sighted per linear kilometer.

Field teams did not conduct dog sight surveys during the Itezi tezhi campaign.

#### 3.2.2 Dog ownership characteristics

During both campaigns, surveyors approached 838 households, of which 671 (80%) respondents gave consent to participate in post-vaccination household surveys [Lusaka: 531/664, 80% and Itezhi tezhi: 140/173, 81%]. Among these 671 households, 264 (39%) reported owning a total of 545 dogs. The 2,894 household members in Lusaka reported owning 200 dogs (HDR: 14.5, 95% CI: 12.7–16.5). The 1,112 household members in Itezhi tezhi reported owning 345 dogs (HDR: 3.2, 95% CI: 1.9–4.1).

**Figure 7:**
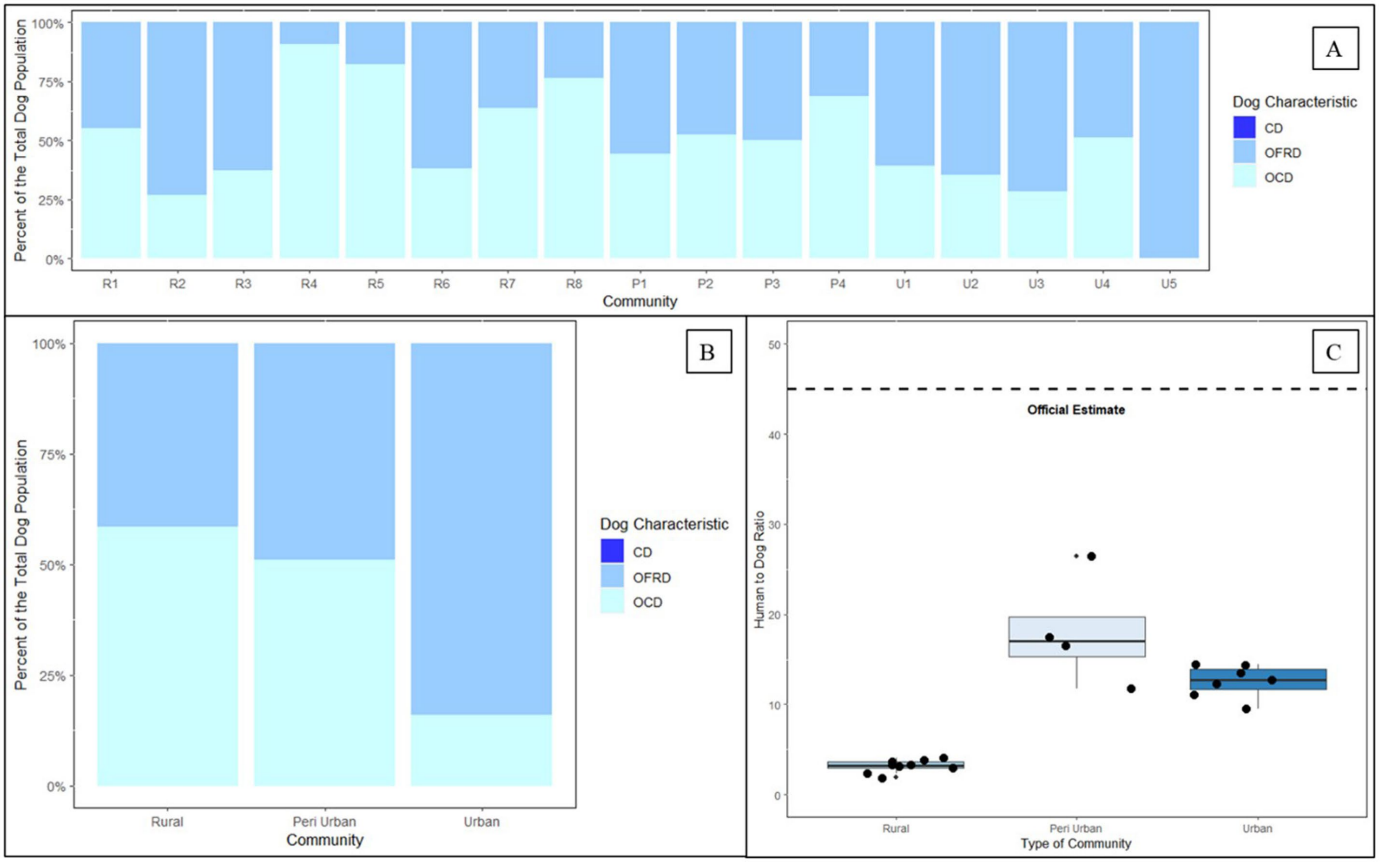
Dog ownership, roaming status, and human to dog ratios derived from two post-vaccination evaluations in Itezhi tezhi (2021) and Lusaka (2022) by type of community. **(A)** Dog ownership and roaming characteristics. **(B)** Dog ownership and roaming characteristics by community type. **(C)** Tukey box and whisker plot of human to dog ratios by community type. CD = community dog; OFRD = owned free-roaming dog; OCD = owned confined dog; R = rural; P = peri-urban; U = urban.

Across all vaccination zones, owners reported most dogs as owned, free-roaming dogs (65%). However, the proportion of free-roaming changed by community type as follows: rural communities—49%, peri-urban communities—50%, and urban communities—82% (Figure 7). Dog ownership and roaming characteristics varied greatly across vaccination zones, ranging from as low as 9% free-roaming to 100%. Based on this analysis, there were no unowned dogs (e.g., “community dogs”) in any vaccination zone (Figure 7).

Among 134 Lusaka dog owners surveyed, 132 (99%) provided their dogs with food, 129 (96%) provided water, 114 (85%) provided shelter, and 85 (63%) provided veterinary care. Overall, 83 (62%) households provided all four services to their dogs and only two (2%) households reported that they provided none of these services to their dogs. Nearly half (42%, 52/123) of Lusaka dog owners reported that they could not easily walk their dog on a leash. Surveyors did not ask these questions in Itezhi tezhi surveys.

In Lusaka, 85 of 530 (16%) households surveyed indicated that they provide some level of care to unowned dogs. Food was the most common offering (*n* = 83), followed by water (*n* = 70), shelter (*n* = 35), and veterinary care (*n* = 19). In Itezhi tezhi, only 10 of 140 (7%) households indicated that they were aware of unowned dogs in their community, with three households reporting seeing unowned dogs in the nearby forests and eight households reporting unowned dogs near rubbish sites.

#### 3.2.3 Public awareness and community sensitization

Among the 134 dog-owning households in Lusaka that provided information on their participation in the vaccination campaign, 32 (24%) respondents had no awareness that the campaign had been conducted and 29 (22%) were made aware of the campaign after the campaign had already started. Overall, nearly half (46%) of dog owners were not reached through pre-campaign sensitization activities.

The most common means of sensitization in Lusaka was through word of mouth, with 48 (36%) dog owners indicating that this was their primary means of awareness. Forty-one dog owners heard about the campaign through megaphones, seven through printed materials, five through radio advertisements, three through healthcare workers, and two through social media.

Among the 130 dog-owning households in Itezhi tezhi that provided information on their participation in the vaccination campaign, 11 (9%) respondents were unaware of the campaign prior to commencement. The most common methods of campaign awareness were the pre-campaign sensitization activities (*n* = 64) and school-based announcements (*n* = 64), followed by community veterinary professionals (*n* = 52) and church (*n* = 20).

#### 3.2.4 Vaccination coverage

In Lusaka, field teams conducted most household surveys within 750 m of the CP sites. The following results are therefore not reflective of coverage among the entire pre-planned vaccination zones. Among the 200 owned dogs reported in the household surveys, 34 were reportedly vaccinated in the past year through private veterinary services, resulting in a pre-campaign dog vaccination coverage of 17% (95% CI: 12–23%). In total, 85 dogs were owner-reported vaccinated through the campaign, resulting in a campaign vaccination coverage of 43% (95% CI: 36–49%). When considering private veterinary and government-sponsored vaccinations within the last year, vaccination coverage was 60% (95% CI: 53–66%).

**Figure 8:**
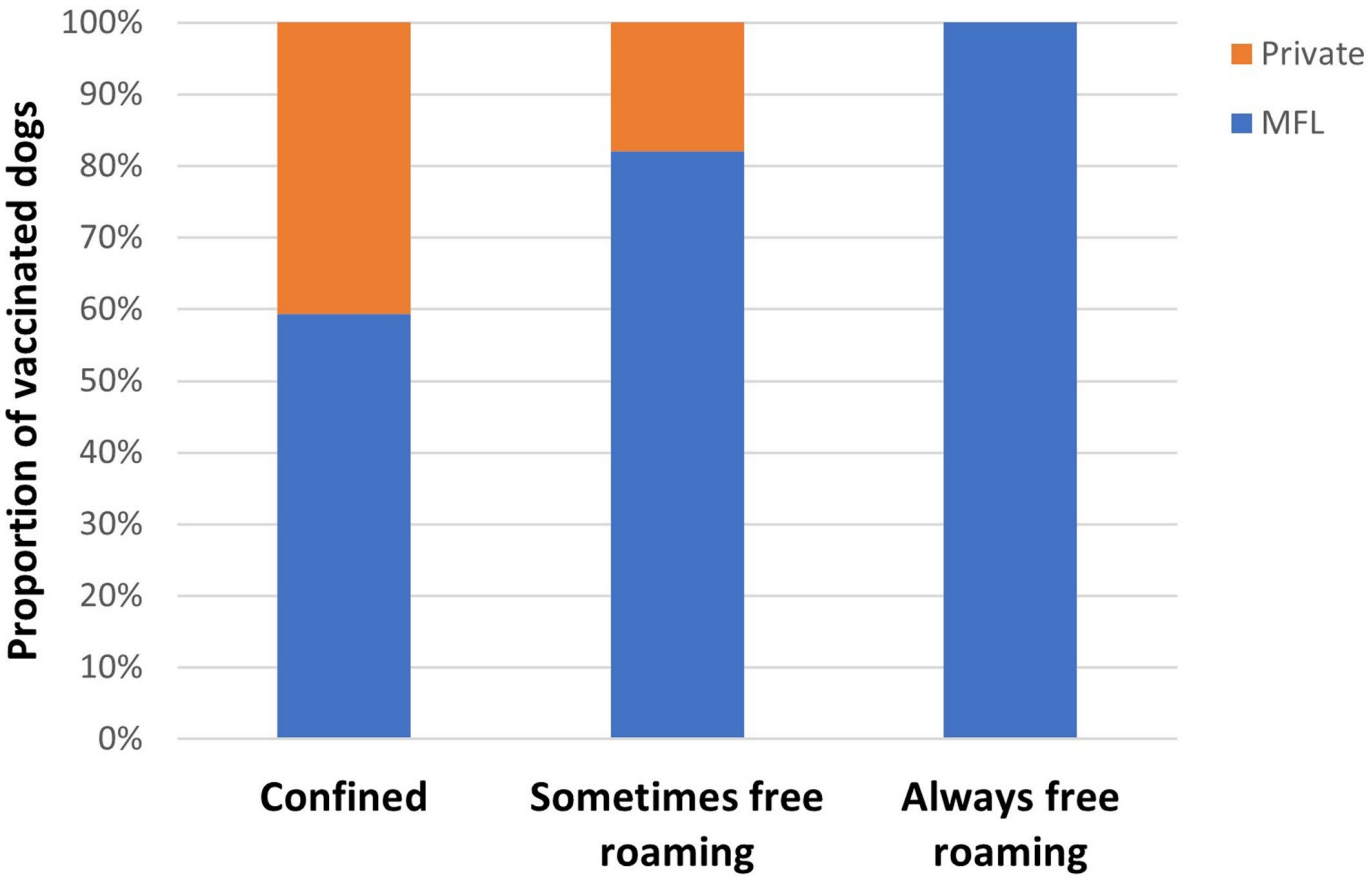
Dog vaccinations reported among household survey respondents by location of vaccination receipt and dog confinement status—Lusaka, Zambia, 2022. Abbreviations: MFL = Ministry of Fisheries and Livestock. Private represents dogs who were reportedly vaccinated in the past year through private veterinary services. MFL represents dogs who were reportedly vaccinated through the MFL-sponsored campaign.

Total pre-adjusted vaccination coverage in Lusaka was highest among confined dogs at 68% (95% CI: 57–77%), followed by “sometimes roaming” dogs at 58% (95% CI: 48–67%), and “always roaming” dogs at 25% (95% CI: 10–50%). Total pre-adjusted vaccination coverage in the past year among all free-roaming dogs (“sometimes roaming” and “always roaming”) was 53% (95% CI: 43–62%). Nearly half (*n* = 24, 41%) of confined dogs were vaccinated through private veterinary services (Figure 8). Fewer free roaming dogs were vaccinated through private veterinary services (*n* = 10, 20%).

Vaccination coverages showed a strong correlation with distance to the CP site in the Lusaka campaign. Dogs residing within 200 m of the CP site had a coverage of 58% compared to 41% when they resided in a home that was >1,500 m from a CP site (Figures 1, 2, 4). When adjusting for the declining vaccination rate related to distance from the CP site, the average vaccination coverage in the vaccination zones was 35%. Additionally adjusting for private vaccination coverage, we estimated the total post-campaign coverage in the vaccination zones at 52%.

In Lusaka, vaccination coverage was positively associated with an increasing perception of the value of the household dog(s). Owners that responded positively to ≥4 dog-perception questions vaccinated 85% of their dogs through government or private veterinary services, compared to 51–59% coverage among households that indicated a lower perceived value for their dogs (*p* = 0.008). Dog owners with higher perceived value for their dogs were also significantly more likely to keep their dogs confined (*p* = 0.001). Surveyors did not ask these questions during the Itezhi tezhi campaign.

Among surveyed households in Itezhi tezhi, 240 dogs were reportedly vaccinated among the 345 total dogs in these households (70% coverage). Reported vaccination coverage was highest in dogs that were allowed to roam freely (74%), compared to 67% coverage among dogs classified as “always confined.” We did not adjust vaccination coverages for the Itezhi tezhi campaign, as there is no routine access to rabies vaccines outside of the annual government vaccination campaigns and field teams conducted a comprehensive door-to-door vaccination strategy.

#### 3.2.5 Willingness to walk assessment

Overall, 95% (62/65) of Lusaka dog owners who attended the vaccination campaign walked or carried their dog to the CP site; only 5% (3/65) of owners drove to the vaccination site. All dog owners surveyed (*n* = 109, 100%) reported that they would walk 100 m to get their dog vaccinated at a government-sponsored dog vaccination campaign (Figure 4, blue line). While approximately 70% (*n* = 78) reported that they would walk up to 1,500 m to get their dog vaccinated, herd immunity (i.e., >70% coverage) was not reached at self-reported walking distances >1,500 m. Willingness to walk was significantly associated with dog owner’s perceived value of their dogs. Households that expressed a strong value (score ≥ 4) were willing to walk an average of 2,889 m, compared to only 1,625 m for households that had a low perceived value for their dogs (score < 1; *p* = <0.001).

Adjusting for observed vaccination coverages at 0–200 m from a CP site (58%) to account for reporting bias in the survey method, herd immunity was not reached with CP vaccination approach at any distance to the CP site and declined rapidly after 1,500 m (Figure 4, red line). Household survey-derived coverages were closely aligned with adjusted reported willingness to walk distances (Figure 4, pink dots).

#### 3.2.6 Barriers to vaccination

Among 37 Lusaka dog owners who did not vaccinate their dogs, the most common reason was that they were unaware of the campaign (*n* = 20, 10% of all dog owning households), followed by respondents indicating their dog was already vaccinated (*n* = 10, 5%), the dog was not home (*n* = 3, 2%), the dog owner was not available (*n* = 3, 2%), long lines (*n* = 1, <1%), the dog was pregnant (*n* = 1, <1%), and the CP was too far (*n* = 1, <1%). Notably, 77 households that did not vaccinate their dog(s) in the campaign provided no excuse.

Among 33 households in Itezhi tezhi that did not vaccinate their dogs, the most common reason was lack of awareness that the campaign was being conducted (*n* = 9, 7% of all dog owning households), followed by inability to restrain their dog, the dog(s) were not home at the time, and they arrived after the campaign had finished for the day (*n* = 5 for each barrier, 4%). Other barriers reported by dog owners included that the dog was too young (*n* = 4), there was no one to take the dog to the vaccination teams (*n* = 4), the dog(s) were too aggressive (*n* = 3), the dog ran away while waiting in line (*n* = 1) and the vaccination teams never knocked on their door (*n* = 1).

#### 3.2.7 Dog population estimation

The HDRs derived from the post-vaccination evaluations (Lusaka: 14.5; Itezhi tezhi: 3.2) estimated far more dogs than previous official estimates using an HDR of 45:1 (Table 1). We observed low HDRs in rural communities, but HDRs were similar in peri-urban and urban communities (Figure 7).

**Table 1:** Estimated dog populations in Lusaka District (A) and Zambia (B).

Population Density (people/km^2^)	Area (km^2^)	Human Population	Total Dog Population	95% CI	Total Dog Density (dogs/km^2^)	HDR	Free Roaming Dog Population	95% CI	Free Roaming Dog Density (dogs/km^2^)
(A): Lusaka District									
>5,000	207	1,957,449	176,775	95,989–257,002	855	11.1	111,189	75,149–143,972	537
500–4,999	179	391,139	6,838	4,824–8,793	38	57.2	2,586	2,225–2,916	14
50–499	57	11,427	981	739–1,224	17	11.6	327	299–357	6
5–49	24	638	383	290–477	16	1.7	127	116–138	5
1–5	5	20	84	64–105	16	0.2	28	26–30	5
0	25	-	-	-	-	-	-	-	-
Total	497	2,360,674	185,061	101,906–267,601	372	12.8	114,257	77,814–147,412	230
(B): Zambia									
>5,000	418	3,619,880	304,044	165,452–441,742	727	11.9	190,925	129,098–247,201	457
500–4,999	4,665	6,465,433	131,744	95,071–167,652	28	49.1	47,747	41,904–53,239	10
50–499	47,095	6,208,771	784,754	592,043–978,006	17	7.9	260,771	238,496–283,947	6
5–49	110,399	2,217,764	1,766,476	1,336,667–2,198,452	16	1.3	583,693	535,249–634,615	5
1–5	14,006	39,584	222,731	168,613–277,140	16	0.2	73,534	67,458–79,931	5
0	577,641	-	-	-	-	-	-	-	-
Total	754,225	18,551,432	3,209,749	2,357,846–4,062,992	4	5.8	1,156,671	1,012,205–1,298,933	2

CI, confidence interval; HDR, human to dog ratio.

**Figure 9:**
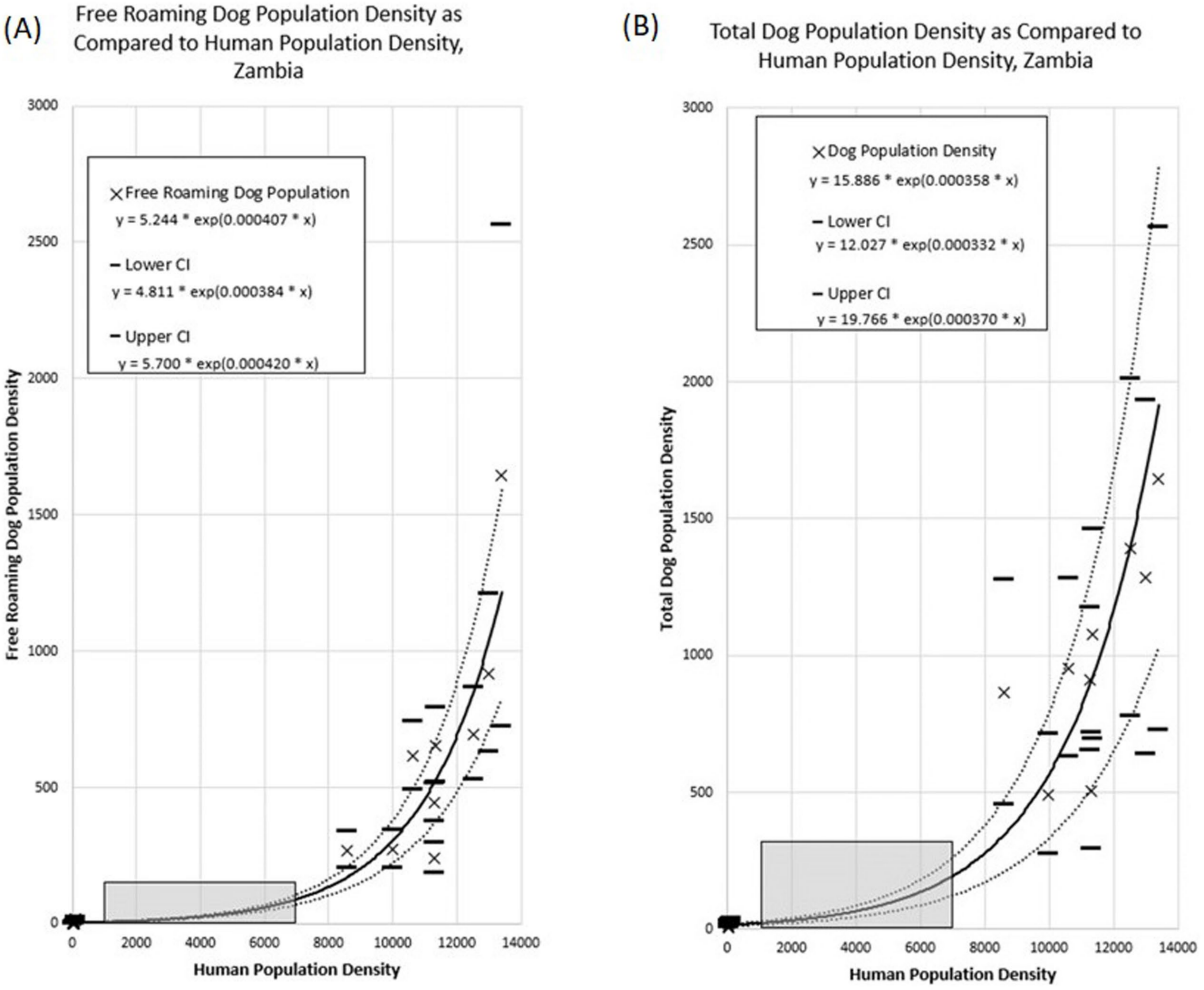
Association between dog population density and human population density across the Itezhi tezhi and Lusaka vaccination zones. **(A)** Free roaming dog population density. **(B)** Total dog population density. Grey box highlights human population densities for which no dog population studies were conducted.

We found a strong correlation between the human density and the dog density across the vaccination zones for both the total dog population (Exponential Function, R^2^ = 0.89) and the free-roaming dog population (Exponential Function, R^2^ = 0.89; Figure 9). The relationship between HDR and human population density showed a significant, but weaker association.

*Total Dog Population Density* = 15.886 * exp(0.000358 * Human Population Density)

*Free Roaming Dog Population Density* = 5.244 * exp(0.000407 * Human Population Density)

Extrapolation of these associations to the entirety of Zambia resulted in a total dog population estimate of 3,209,749 (95% CI: 2,357,847–4,062,992) and a free-roaming dog population estimate of 1,156,671 (95% CI: 1,012,205–1,298,933; Table 1). The largest dog populations reside in rural communities (62%), followed by peri-urban communities (24%), with only 14% of the dog population residing in urbanized communities.

## 4 Discussion

Despite the MFL conducting government-sponsored rabies vaccination campaigns since at least 2013, rabies remains enzootic in dog populations across Zambia. While there are examples of rabies vaccination programs that were implemented with limited knowledge of the dog population, rabies persistence in the face of these programs is a strong indication of chronic under vaccination. In these situations, studies such as the one described in this report are necessary to revise vaccination approaches, improve owner participation, and eventually reach herd immunity. To-date, this is the largest dog population and vaccination program evaluation that has been conducted in Zambia. The inclusion of both rural, peri-urban, and urban communities in this analysis has shed light on unique challenges that these communities and their respective local governments face to reach anti-rabies herd immunity.

Prior to this analysis, Zambia MFL relied on antiquated dog population estimates, of which the estimation methods were unknown to vaccination program managers. Relying on historical estimates of 45 people per dog, vaccination programs procured vaccines with the goal of reaching up to 250,000 dogs annually. However, as demonstrated in this report, there are likely far more dogs in Zambia than previously considered. Further, the distribution of these dogs varies by community-type, with particularly high exponential association between dog density and human density. We found most dogs in rural, low-population density communities, where it is questionable if dog-mediated rabies can persist at enzootic levels. Previous studies have shown that vaccination programs that target rural communities are more costly, largely due to logistical expenses associated with transporting vaccines and people to sparsely populated areas over long distances (11, 16, 22, 23). While there is still debate regarding the role of rural communities in the maintenance of rabies virus transmission, the results here suggest that prioritizing peri-urban and urban communities for large-scale dog vaccination programs could reduce the target dog population by more than half (24–26). While it is idealistic to attempt to rabies-vaccinate all dogs in a country, low- and middle-income countries must prioritize limited resources so that they are maximally effective. The results here provide some insight into how a strategic vaccination campaign could be conducted among higher-density free-roaming dog populations. The dog population estimates obtained from this study align with many other studies with published HDRs (12, 14, 27). However, it should be noted that dog estimation methods are fraught with error and there is much variation in dog population estimates between communities and across countries.

In addition to a much higher dog population than previously considered, we identified numerous barriers to reaching herd immunity, with unique obstacles noted in rural and urbanized communities. In the rural community of Itezhi tezhi, herd immunity was achieved, whereas vaccination coverages in the urban communities of Lusaka only reached 35%. One of the primary reasons for the lower coverage was likely in the campaign design. Itezhi tezhi utilized a hybrid campaign of CP sites followed by a door-to-door approach. Further, Itezhi tezhi had a large investment in pre-campaign awareness and App-derived daily guidance for moving vaccination teams. As a result, only 9% of dog owners claimed they were unaware of the campaign prior to commencement. Inability to reach the campaign was rarely cited as a barrier in Itezhi tezhi. In contrast, pre-campaign sensitization in Lusaka was much more difficult as an urbanized community. Three-fold more dog owners were unaware of the campaign and far more dog owners indicated lack of awareness as a primary barrier to vaccination. Social means of pre-vaccination sensitization may prove fruitful in Lusaka, and considerations for alternative sensitization programs may be warranted.

We explored barriers to vaccination in more detail during the Lusaka campaign. This included exploration of owners perceived value of their dogs and their willingness to walk with their dogs to CP sites. These results cast doubt over the effectiveness of CP vaccination approaches in urbanized settings like Lusaka. Notably, over half of dog owners indicated that they could not easily walk their dog on a leash. Further, the average distance an owner reported they were willing to walk to a CP site was only 1750 m. There was a significant difference between the distance owners claimed they would walk and how far they actually walked during the campaign. As such, social desirability bias likely influenced the dog owner’s self-reported willingness to walk distances. When attempting to adjust the survey results to account for this bias, the highest coverage likely to be achieved through a CP approach is about 60%, and this would only be if all dog owners had a campaign located within 500 m of their residence. To put this into the context of Lusaka District, which is approximately 360 square kilometers, over 450 CP sites would need to be conducted across the city to accommodate the dog owner’s willingness to walk 500 m for vaccination services.

Several factors explored in this study appear to counteract the low willingness to walk. Notably, dog owners who reported a higher perceived value for their dogs were willing to walk nearly twice as far to get them vaccinated. Additionally, the household survey-reported coverage among these dog owners was 88% (pre-adjustment). These findings suggest that programs that improve the human-animal bond and owner education would likely make CP vaccination approaches more successful. The CP approach is the lowest cost of the conventional vaccination approaches and is by far the easiest to logistically implement. More exploration of impactful and cost-effective methods to improve human-animal bond could have significant impact on the success of rabies elimination in urbanized communities.

Another unique finding in the urbanized communities of Lusaka was the relatively high proportion of dog owners who utilized private veterinarians for vaccination services. Private veterinarians can be an important stakeholder in achieving herd immunity, but many countries struggle to regulate private veterinary practices and track their rabies vaccination activities. Furthermore, offering vaccines freely through the government and at-cost from private veterinary practices can lead to confusion among dog owners as to how and when to vaccinate their dogs and can cause contention between public and private veterinary professionals.

The results from the Lusaka evaluation may shed light on an interesting approach that could satisfy all partners: government, private veterinarians, and dog owners. While private veterinary services were commonly utilized among surveyed dog owners, the population of dogs that were provided this care were those that were always under owner confinement. Always-confined dogs likely play a very minimal role in the maintenance of rabies virus transmission. Conversely, dog owners who allowed their dogs to roam freely were more likely to utilize the free government-provided rabies vaccines. The goal of private veterinarians should be the health of the individual animal they are treating, whereas the goal of a government-sponsored rabies campaign should be to reach herd immunity in the free-roaming dog population. Interestingly, the survey results here strongly suggest that there is an important role for private veterinarians to vaccinate primarily owned always-confined dogs. In theory, this could allow government vaccination services to focus on the free-roaming dog population. Development of a regulatory scheme in which private veterinarians have a clear role in rabies vaccination, ensure that they use quality vaccines, and track their vaccine usage in a way that can be reported to government officials could be a successful strategy in urbanized communities.

The use of the WVS App enabled study coordinators to track key operational factors associated with dog vaccination campaigns, including post-vaccination evaluations and dog population estimations. Using digital tools to aid in dog vaccination campaigns is becoming common-place and is now recommended by international agencies such as the World Organization for Animal Health and World Health Organization. However, studies have shown that one of the primary benefits of utilizing digital data collection tools is for the implementation of the Vaccinate-Assess-Move methodology (12, 28). This approach relies on the routine (e.g., daily) collection of vaccination data, review by a managerial team, and re-direction of vaccination teams based on their real-time performance. This approach was implemented in Goa, India, which has now been declared free from dog-mediated human rabies deaths (29). The WVS App has been used in numerous other settings to implement this vaccination approach, with multiple publications and reports suggesting that it can rapidly increase dog vaccination coverages (12). Vaccinate-Assess-Move requires additional management support for daily data review and team direction, but in communities with persistent rabies cases due to low herd immunity, it is a proven-successful method and one that could be implemented in Zambia.

This paper describes an operational research study that evaluated multiple components of two complex dog vaccination campaigns, resulting in several noteworthy limitations. First, key data were missing in non-negligible quantities, including dog roaming characteristics and their participation in the dog vaccination campaign. We addressed this missing data through an assumption that all dogs in the household were treated in the same manner. Therefore, if data were available for at least one dog, we were able to apply a roaming and vaccination status. We feel this approach is reasonable but is prone to error. Additionally, household survey data, particularly regarding self-reported vaccination coverage and dog ownership characteristics, may be prone to recall bias, potentially leading to overreporting of vaccination coverage. Social desirability bias may have influenced responses to questions about willingness- to-walk dogs to vaccinate sites, with participants potentially overstating their commitment to walking behaviors. Second, local coordinators in Lusaka did not select the vaccination zones at random, which could result in biased conclusions regarding the representativeness and generalizability of the vaccination coverage and dog populations in these areas. Pre-study HDR estimates were 45:1, yet this value was 3-times lower in the selected vaccination zones. With the methods used for this study, we are not able to conclude if the vaccination zones selected have an abnormally low HDR, leading to inflated extrapolated dog population estimates. Lastly, the study zones selected represent two extremes of Zambia’s communities: a very low-density community and a very high-density community. This resulted in a large data-gap for communities with human densities between 100 and 7,000 people per square kilometer (Figure 9, gray box). Future studies should prioritize communities like these to determine if the trends identified in this study are accurate.

## 5 Conclusion

Zambia has experienced persistent rabies deaths in people and animals because of chronically low dog vaccination coverage despite years of governmental support to offer dog rabies vaccines to affected communities. In the face of persistent rabies deaths, studies such as these can offer evidence-based solutions to improve dog vaccination coverage. As is commonly reported in rabies endemic countries, one primary cause of persistent rabies in Zambia is a historical dog population estimate that is 3–5 times lower than what this study identified. While a detailed understanding of the dog population is not always necessary to achieve elimination, these studies should be conducted when there are persistent rabies cases despite active vaccination programs. Improving rabies herd immunity in Zambia may be challenging, particularly in more urbanized settings. Future campaigns must focus on community sensitization and address the short distances that many dog owners are able (or willing) to walk with their dogs. Future campaigns could prioritize a longer lead-in time for sensitization efforts to build awareness within communities, coupled with a targeted advertising and media plan. This could include leveraging local influencers, engaging with community leaders, and using a variety of media channels (e.g., radio, social media, posters in high-traffic areas) to communicate the importance of vaccination and the availability of convenient services, ensuring that dog owners are both informed and motivated to act. Investment in programs that improve dog owners’ perceived value of their pets would likely be dually beneficial to rabies elimination in Zambia. First, through a likely increase in dog owner’s participation in CP campaigns and secondly, through a cultural shift towards ownership of confined dogs which would likely result in increased utilization of private veterinarians for vaccine services. Private veterinary services clearly play an important role in the vaccination of owned always-confined dogs in Zambia; however, programs must be developed to ensure that private veterinarians use best-practices and share vaccination data with government officials. Herd immunity can be reached in Zambia, as demonstrated in Itezhi tezhi, but unique community-based approaches will need to be developed. One-size-fits-all vaccination will likely not lead to successful outcomes. With the improved knowledge of dog and ownership characteristics, Zambia is well-prepared to design more effective vaccination campaigns as they expand their rabies elimination program. Future campaigns should strengthen community sensitization, invest in initiatives that increase dog owners’ perception of their pets, and implement adaptable vaccination strategies (e.g., combining CP with door-to-door efforts) that engage both public and private veterinary services.

## Data Availability

The original contributions presented in the study are included in the article/Supplementary material, further inquiries can be directed to the corresponding author.

## Footnotes

^1^ https://maps.google.com
