# Supplementary material for: Rabies vaccinations at the rural–urban divide: successes and barriers to dog rabies vaccination programs from a rural and urban campaign in Zambia
Source: Front Vet Sci. 2025 Jan 20;11:1492418. doi: 10.3389/fvets.2024.1492418 (PMC11789232; doi:10.3389/fvets.2024.1492418)
Supplement: Supplementary file 1 [file Table_1.DOCX]

**Supplemental Table 1.** Community and human population characteristics by vaccination zone — Lusaka, Zambia, 2022 and Itezhi tezhi, Zambia, 2021

| **Campaign** | **Vaccination Zone** | **Urbanicity Level*** | **Total Human Population** | **Community (km^2^)** | **SRS Transect Human Population Density** |
| --- | --- | --- | --- | --- | --- |
| Lusaka | Plainview | High | 70,623 | 7.1 | 9,987 |
|  | Kanyama | High | 80,229 | 7.1 | 11,345 |
|  | Twashuka | High | 56,298 | 5.3 | 10,615 |
|  | Misisi | High | 30,361 | 3.5 | 8,586 |
|  | Matero | High | 57,356 | 4.4 | 12,975 |
|  | Chibolya | High | 19,942 | 1.8 | 11,279 |
|  | Lillanda | High | 199,752 | 18 | 11,297 |
|  | Mandevu | High | 201,351 | 15.0 | 13,396 |
|  | Kabannana | High | 210,147 | 16.8 | 12,509 |
| Itezhi tezhi | A | Low | 329 | 11.5 | 29 |
|  | B | Medium | 627 | 7.9 | 79 |
|  | C | Low | 272 | 8.8 | 31 |
|  | D | Low | 250 | 5.3 | 47 |
|  | E | Low | 310 | 9.7 | 32 |
|  | F | Medium | 266 | 5.3 | 50 |
|  | G | Medium | 702 | 6.2 | 114 |
|  | H | Low | 318 | 7.0 | 45 |

*Urbanicity levels were defined based on population density as follows: urban = areas with population density of at least 500 people/km^2^; peri-urban = areas with population density 50–499 people/km^2^; rural = areas with fewer than 50 people/km^2^.
