# Supplementary material for: Rabies vaccinations at the rural–urban divide: successes and barriers to dog rabies vaccination programs from a rural and urban campaign in Zambia
Source: Front Vet Sci. 2025 Jan 20;11:1492418. doi: 10.3389/fvets.2024.1492418 (PMC11789232; doi:10.3389/fvets.2024.1492418)
Supplement: Supplementary file 2 [file Table_2.DOCX]

**Mass rabies dog vaccination evaluation:**

**Knowledge, attitudes, and practices survey**

July 2022

Lusaka, Zambia

| **Introduction** | |
| --- | --- |
| **To the interviewee:** “Thank you for being willing to participate in this survey. I am going to start by asking you basic questions about yourself to get to know you better. Your responses will help us better understand people’s knowledge of rabies in Lusaka. It will also help us identify barriers that make it difficult for people to proactively vaccinate their dogs to prevent exposures. Please note that your name and any other identifying information will not be collected during this survey. Participation is entirely voluntary and you may tell us to stop the survey at any time for any reason. [provide consent leaflet] | |
| 1. Indicate response below:   I am 18 years of age or older   - Consent given - Consent not given - Adult respondent not home/available - Household missing - Household abandoned | 🡪Save and begin the interview  🡪Save and go to the next selected household  🡪Save and go to the next selected household and return at a later time  🡪Save and go to the next selected household  🡪Save and go to the next selected household |

| **I. Interviewer Information** | | |
| --- | --- | --- |
| **No.** | **Question** | **Response** |
| 101 | Name of data collector | ____________________________________ |
| 102 | Date (DD/MM/YYYY): | ___ ___/ ___ ___/ ___ ___ ___ ___ |
| 103 | Evaluation Zone | ___ ___ |

| **II. Demographics** | | |
| --- | --- | --- |
| **No.** | **Question** | **Response** |
| 201 | Is the respondent head of household? | □ Yes □ No |
| 202 | What is your age? | (#) ___________ years |
| 203 | What is your gender? | **□** Male **□** Female **□** Other |
| 204 | How many people live in your household (including self)? | (#) _______________ |
| 205 | How many people living in your household are under 18 years of age? | (#) _______________ |

| **III. Knowledge: Rabies and rabies vaccination** | | | | |
| --- | --- | --- | --- | --- |
| **No.** | | **Question** | **Response** | |
| 301 | | Of the following, which disease do you most fear getting: *Select one* | **□** Malaria **□** HIV/AIDs **□** Rabies  **□** Tuberculosis **□** Influenza **□** Don’t know  **□** Other:_____________ | |
| 302 | | Before this survey, had you ever heard of a disease called ‘rabies’? | **□** Yes  **□** No **□** Don’t know | - Skip to 401 |
| 303 | | Do you know anyone in your community who has ever died from a disease called ‘rabies’? | **□** Yes  **□** No **□** Don’t know | |
| 304 | | How severe is the disease called ‘rabies’? | **□** Mild **□** Somewhat severe  **□** Very severe, but recoverable  **□** Very severe, fatal  **□** Don’t know **□** Decline to answer | |
| 305 | | How do humans get rabies from an infected animal?  *Read choices. Mark all that apply* | **□** Bite **□** Scratch **□** Observing the animal  **□** Touching the animal **□** Contact with blood  **□** Contact with saliva **□** Contact with urine/feces  **□** Don’t know **□** Decline to answer  **□** Other: _______________________________ | |
| 306 | | On a scale of 1 to 5, with 1 being little to no risk of rabies from that animal, list the rabies risk of each animal | **□** Dogs:  **□** Cats:  **□** Livestock:  **□** Bats:  **□** Rodents | 1 2 3 4 5  1 2 3 4 5  1 2 3 4 5  1 2 3 4 5  1 2 3 4 5 |
| 307 | Do you think it is important that a dog be vaccinated against rabies? | | **□** Yes **□** No 🡪 Skip to 309 | |
| 308 | How often should a dog be vaccinated against rabies? | | **□** At least once a year **□** Every other year  **□** 2-3 times per lifetime **□** Once in a lifetime | |
| 309 | Do you think the rabies vaccine poses any risks to a dog? | | **□** Yes **□** No 🡪 Skip to 401 | |
| 310 | What are the risks to dogs from being vaccinated against rabies?  *Don’t read. Assessor should listen to the respondent and then mark all that apply.* | | **□** Produces anxiety in the dog  **□** Produces bleeding in the dog  **□** Can cause rabies  **□** Can cause other infectious diseases  **□** Can cause diarrhea/vomiting  **□** Can cause sterility  **□** Will become bad guard dog  **□** Can cause other side effects  **□** Death **□** I don’t know **□** No response  **□**Other: _____________________________ | |

|  | **IV. Bite rates** | | | | | | | | | | | | | | |
| --- | --- | --- | --- | --- | --- | --- | --- | --- | --- | --- | --- | --- | --- | --- | --- |
| **No.** | |  | | | **Question** | | | | | | | **Response** | | | |
| 401 | |  | | | **In the past year**, have you **or anyone in your household** been bitten or scratched by an animal? | | | | | | | - Yes - No | - Skip to 501 | | |
| 402 | |  | | | Please provide information on each bite or scratch event that has occurred among members of your household. *{Complete one line for every person bitten/scratched and Repeat for every bite event (e.g., if a person had multiple bites during the year, each bite event should be recorded separately)}* | | | | | | | | | | |
| **a. Victim Age** | | | **b. Year and Month of bite** | **c. Type of Animal** | | **d. Animal was:**  **1. Mine**  **2. Neighbors**  **3. Unknown** | **e. Where on the body did the bite/scratch occur?** | **f. Type of exposure** | **g. Did the animal die within 10 days of biting?** | **h. Did you/they seek medical care?**  **Yes-med 🡪 403**  **Yes-trad 🡪 404**  **No🡪 404** | **i. Did the person experience any complications/infections related to the bite wound?** | | | **j. Is this person still alive?** | |
| **1.** | | |  | Dog  Cat  Other | | Mine  Neighbors  Unknown | Head/Neck  Chest  Arm/ Hand  Leg/ Foot  Unknown | Bite  Scratch | ○ Yes  ○ No  ○ I don’t know | ○ Yes - medical  ○ Yes - traditional  ○ No  ○ I don’t know | ○ Yes  ○ No  ○ I don’t know | | | ○ Yes  ○ No  ○ I don’t know | |
| **2.** | | |  | Dog  Cat  Other | | Mine  Neighbors  Unknown | Head/Neck  Chest  Arm/ Hand  Leg/ Foot  Unknown | Bite  Scratch | ○ Yes  ○ No  ○ I don’t know | ○ Yes - medical  ○ Yes - traditional  ○ No  ○ I don’t know | ○ Yes  ○ No  ○ I don’t know | | | ○ Yes  ○ No  ○ I don’t know | |
| **3.** | | |  | Dog  Cat  Other | | Mine  Neighbors  Unknown | Head/Neck  Chest  Arm/ Hand  Leg/ Foot  Unknown | Bite  Scratch | ○ Yes  ○ No  ○ I don’t know | ○ Yes - medical  ○ Yes - traditional  ○ No  ○ I don’t know | ○ Yes  ○ No  ○ I don’t know | | | ○ Yes  ○ No  ○ I don’t know | |
| **4.** | | |  | Dog  Cat  Other | | Mine  Neighbors  Unknown | Head/Neck  Chest  Arm/ Hand  Leg/ Foot  Unknown | Bite  Scratch | ○ Yes  ○ No  ○ I don’t know | ○ Yes - medical  ○ Yes - traditional  ○ No  ○ I don’t know | ○ Yes  ○ No  ○ I don’t know | | | ○ Yes  ○ No  ○ I don’t know | |
| **5.** | | |  | Dog  Cat  Other | | Mine  Neighbors  Unknown | Head/Neck  Chest  Arm/ Hand  Leg/ Foot  Unknown | Bite  Scratch | ○ Yes  ○ No  ○ I don’t know | ○ Yes - medical  ○ Yes - traditional  ○ No  ○ I don’t know | ○ Yes  ○ No  ○ I don’t know | | | ○ Yes  ○ No  ○ I don’t know | |
|  | |  | | |  | | | | | | | | | | |
| 403 | |  | | | If the victims sought medical care, please fill out the table: | | | | | | | | | |  |
|  | \| **Victim (as assigned above)** \| **a. How many days occurred between when they were bitten on this occasion and medical care was sought?** \| **b. Did they receive rabies vaccine?** \| **c. How many times/days (including the first visit where vaccine was received) did they return to a clinic to receive a dose of rabies vaccine?**  **Options: 1, 2, 3, 4, Unknown** \| **d. Did the person receive rabies immuno-globulin?** \| **e. If the vaccine was not received, why was the vaccine not received?**   1. **Did not think it was necessary** 2. **Lack of nearby facilities to provide treatment** 3. **Lack of trained personnel at facilities** 4. **Lack of vaccines at facility** 5. **No means of transportation** 6. **Cost of vaccine** 7. **Would have to miss work** 8. **Other, specify** \| **f. If not all (5) doses were received, why were not all doses received?**   1. **Did not think it was necessary** 2. **Adverse event occurred** 3. **Forgot** 4. **Dog was tested negative** 5. **Dog was observed in quarantine** 6. **Not pleased with original experience at facility** 7. **Distance to facility too far** 8. **Would have to miss work** 9. **Other, specify** \| \| --- \| --- \| --- \| --- \| --- \| --- \| --- \| \| **1.** \|  \| ○ Yes  ○ No  ○ I don’t know \|  \| ○ Yes  ○ No  ○ I don’t know \|  \|  \| \| **2.** \|  \| ○ Yes  ○ No  ○ I don’t know \|  \| ○ Yes  ○ No  ○ I don’t know \|  \|  \| \| **3.** \|  \| ○ Yes  ○ No  ○ I don’t know \|  \| ○ Yes  ○ No  ○ I don’t know \|  \|  \| \| **4.** \|  \| ○ Yes  ○ No  ○ I don’t know \|  \| ○ Yes  ○ No  ○ I don’t know \|  \|  \| \| **5.** \|  \| ○ Yes  ○ No  ○ I don’t know \|  \| ○ Yes  ○ No  ○ I don’t know \|  \|  \| | | | | | | | | | | | | | |  |

| 404 | If the victim did not seek medical care, please fill out this table:  *Don’t read. Assessor should listen to the respondent and then mark all that apply.* |
| --- | --- |
| \| **Victim (as assigned above)** \| **Why did they not seek medical care for this bite at a healthcare facility?**   1. **Did not think it was necessary** 2. **Lack of nearby facilities to provide treatment** 3. **Lack of trained personnel at facilities** 4. **Lack of vaccines at facility** 5. **No means of transportation** 6. **Cost of vaccine** 7. **Would have to miss work** 8. **Use of traditional medicine** 9. **Other, specify** \| \| --- \| --- \| \| **1.** \|  \| \| **2.** \|  \| \| **3.** \|  \| \| **4.** \|  \| \| **5.** \|  \| | |

| **VI. Dog ownership** | | | | | | |  |
| --- | --- | --- | --- | --- | --- | --- | --- |
| **No.** | **Question** | | | **Response** | | |  |
| 501 | Does your household currently own any dogs? | | | **□** Yes **□** No | | |  |
| 501a | Do you provide care for any dogs that you do **NOT** own?  *Mark all that apply* | | | **□** No **□** Food **□** Water **□** Shelter  **□** Veterinary care, including rabies vaccination  **□** Other: ___________________________  **□** No response | | |  |
| 501b | For interviewer only: Does the interviewee say that they have dogs (501) and/or that they provide veterinary care (501a)? | | | **□** Yes **□** No 🡪 Skip to 509 | | |  |
| 502 | How many dogs are owned by you or other people in your household? | | | ____________________ dogs | | |  |
| 503 | What level of care does your household provide for your dogs?  *Select all that apply* | | | **□** None **□** Food **□** Water  **□** Shelter **□** Veterinary care  **□** No response  **□** Other: _____________________ | | |  |
| *For questions 504 to 508, please state whether you agree or disagree with the following statements (DOG OWNERS ONLY):* | | | | | | | |
| 504 | | My dogs are a part of my family. | | | | **□** Agree **□** Disagree **□** No opinion | |
| 505 | | If my dog were to die, it would be easy to replace him/her. | | | | **□** Agree **□** Disagree **□** No opinion | |
| 506 | | I feel affection for my dogs. | | | | **□** Agree **□** Disagree **□** No opinion | |
| 507 | | My dog is accustomed to walking on a leash. | | | | **□** Agree **□** Disagree **□** No opinion | |
| 508 | | Other people (family, friends, veterinarians) have encouraged me to vaccinate my dogs | | | | **□** Agree **□** Disagree **□** No opinion | |
|  | |  | | | |  | |
| 509 | | | Did your household own any dogs that died **in the past year?** | | **□** Yes **□** No 🡪 Skip to 601 | | |
| 510 | | | How many dogs from your household died in the past year? | | ____________________ dogs | | |
| 511 | | | *For dogs who died,* fill out the following table: | | | | |
| \| **Dog** \| **Month of death** \| **Cause of death**   1. **Hit by car** 2. **Poisoned** 3. **Rabies** 4. **Other disease/illness** 5. **Killed by person** 6. **Age related** 7. **I don’t know** 8. **Other, specify** \| **In the past year,** did any of those dogs **die** shortly after displaying any of the following symptoms? \| \| \| \| \| \| \| --- \| --- \| --- \| --- \| --- \| --- \| --- \| --- \| --- \| \| **Hypersalivation** \| **Aggression** \| **Biting (people or animals)** \| **Difficulty walking** \| **Change in bark** \| **If bit people, Number of people bitten** \| \| 1 \|  \|  \| ○ Yes ○ No \| ○ Yes ○ No \| ○ Yes ○ No \| ○ Yes ○ No \| ○ Yes ○ No \|  \| \| 2 \|  \|  \| ○ Yes ○ No \| ○ Yes ○ No \| ○ Yes ○ No \| ○ Yes ○ No \| ○ Yes ○ No \|  \| \| 3 \|  \|  \| ○ Yes ○ No \| ○ Yes ○ No \| ○ Yes  ○ No \| ○ Yes ○ No \| ○ Yes ○ No \|  \| \| 4 \|  \|  \| ○ Yes ○ No \| ○ Yes ○ No \| ○ Yes ○ No \| ○ Yes ○ No \| ○ Yes ○ No \|  \| \| 5 \|  \|  \| ○ Yes ○ No \| ○ Yes ○ No \| ○ Yes ○ No \| ○ Yes ○ No \| ○ Yes ○ No \|  \| \| 6 \|  \|  \| ○ Yes ○ No \| ○ Yes ○ No \| ○ Yes ○ No \| ○ Yes ○ No \| ○ Yes ○ No \|  \| \| 7 \|  \|  \| ○ Yes ○ No \| ○ Yes ○ No \| ○ Yes ○ No \| ○ Yes ○ No \| ○ Yes ○ No \|  \| | | | | | | | |

| **VII. Campaign awareness and barriers to dog rabies vaccination** | | | | | | |
| --- | --- | --- | --- | --- | --- | --- |
| **No** | | **Question** | | **Response** | | |
| 601 | | Did you know that a dog rabies vaccination campaign was taking place in your community over the past 2 weeks? | **□** Yes **□** No 🡪 Skip to 701 or end survey | | |  |
| 602 | | What type of dog vaccination activity did you see in your community over the past 2 weeks?  *Read off and select all that apply* | **□** People using nets to catch and vaccinate dogs  **□** Vaccinators setting up clinics  **□** Vaccinators came to my door to offer vaccination  **□** I saw dogs in my community with paint marks on them | | |  |
| 603 | | How and when (before, during, after) did you hear about the campaign? | \|  \| Before \| During \| After \| \| --- \| --- \| --- \| --- \| \| Print media (newspapers, posters, leaflets) \|  \|  \|  \| \| Megaphone \|  \|  \|  \| \| Radio \|  \|  \|  \| \| Friend/neighbor \|  \|  \|  \| \| Health care worker \|  \|  \|  \| \| Other, specify____________ \|  \|  \|  \| | | |  |
| End of survey for all households who do not own dogs.  **The following questions should only appear if ‘Yes’ to 501b (i.e., ‘Yes’ to 501 AND/OR 501a).** | | | | | | |
| 701 | | Do you prefer to have your dog vaccinated during a campaign or at the vet’s office/clinic?  **□** No preference 🡪 skip to 704  **□** Would not vaccinate 🡪 skip to 704 | | | | |
|  | | **□** Government campaign [*for assessor: free vaccine*]  **□** Veterinary clinic campaign [*for assessor: reduced-price vaccine*]  **□** Veterinarian office/clinic 🡪 skip to 703 | |  | | |
| 702 | | What are reasons you prefer to have your dog vaccinated at a campaign?  *Don’t read. Assessor should listen to the respondent and then mark all that apply.* | | **□** There are no veterinary offices/clinics near my house  **□** The campaign is free or reduced price  **□** The campaign is closer to my house/vet office is far from my house  **□** The campaign takes less time/vet office takes more time  **□** Bad experience at vet office  **□** Do not like veterinarian  **□** The staff is better qualified at the campaign  **□** The vaccine quality is better at the campaign  **□** Transporting my dog to the campaign is easier  **□** Other: ___________________________ | | |
| 703 | | What are reasons you prefer to have your dog vaccinated at a veterinarian office/clinic?  *Don’t read. Assessor should listen to the respondent and then mark all that apply.* | | **□** The campaign is not reliable  **□** The campaign does not give me enough notice that they are coming  **□** The staff is better qualified at the vet office  **□** The staff is more attentive/warm at the vet office  **□** The vet office is closer to my house  **□** I can choose my own hours at the vet office/timing of the campaign is not convenient  **□** The campaign does not occur on a regular schedule  **□** The vaccine quality is better at the vet office  **□** The vet office is cleaner  **□** The campaign is more likely to reuse needles  **□** Transporting my dog to the vet is easier  **□** It is easier to avoid dog fights at the vet office/the risk of dog fight or bite at the campaign is high  **□** Bad experience at previous campaign  **□** Other: ___________________________ | | |
| 704 | | If rabies vaccine were offered to your dog, would you or someone from your household be willing and able to travel 1 km with your dog to a vaccination clinic? | | | | |
|  | **□**Yes **□**No | | | |  |  |
|  | A.1 If *Yes,* Would you travel 1.5 km? | | | B.1 If *No,* Would you travel 500 m? |  |  |
|  | **□** Yes **□** No -> Skip to 705 | | | **□** Yes -> Skip to 705 **□** No |  |  |
|  | A.2 If *Yes,* Would you travel 2 km? | | | B.2 If *No,* Would you travel 250 m? |  |  |
|  | **□** Yes **□** No -> Skip to 705 | | | **□** Yes -> Skip to 705 **□** No |  |  |
|  | A.3 If *Yes,* Would you travel 3 km? | | | B.3 If *No,* Would travel 100 m? |  |  |
|  | **□** Yes **□** No -> Skip to 705 | | | **□** Yes -> Skip to 705 **□** No |  |  |
|  | A.4 If *Yes,* If you are willing and able to walk more than 3 km, please specify the amount you are willing to travel:  _____________ km | | | B.4 If *No*, if you are unable or unwilling to travel 100 m, please specify the amount you are willing to travel:  ______________ m |  |  |
| ***If the campaign was a central point:*** | | | | | | |
| 705 | | Did you or someone from your household bring any dogs (both owned and/or unowned) to the vaccination campaign? | | **□** Yes  **□** No 🡪 skip to 711  **□** I don’t know 🡪 skip to 712 | | |
| 706 | | Which location of the campaign did you go to? | | **□** Kanyama old/new—Masauuko market  **□** Kanyama old/new—council offices  **□** Kanyama old/new—Twashuka ground  **□** Chawama—city council office/market  **□** Matero—welfare hall  **□** Misisi—misisi ground  **□** Johnlaingh —Johnlaingh basic school  **□** Chilenje —Chilenje ground  **□** Jack Compound—Kamulanga school  **□** Kamwala— Kamwala basic school  **□** Libala—Katungu  **□** Kabwata—Kabwata basic school  **□** Chibolya—water trust  **□** Chibolya—TBD  **□** Other: _____________________ | | |
| 707 | | How long did it take you to get to the campaign from your house? | | _____________ mins | | |
| 708 | | How did you get your dog to the campaign? | | **□** I walked it  **□** I carried it  **□** I dragged it  **□** I took it by vehicle  **□** I took it by bicycle  **□** Other: ______________________ | | |
| 709 | | How long did you/your household member have to wait in line at the campaign? | | _____________ min | | |
| 710 | | Was your dog bitten while standing in line at the campaign? | | **□** Yes **□** No | | |
| 711 | | *If no to 705,* Why did you not go to the campaign?  *Don’t read. Assessor should listen to the respondent and then mark all that apply.* | | **□** I did not know about it  **□** It is not important to vaccinate against rabies  **□** I was unavailable or had to work at the time of the campaign  **□** I could not find my dogs  **□** The campaign was far from my house  **□** I saw long lines at the campaign  **□** Transporting my dogs to the campaign is difficult  **□** I do not trust the vaccine  **□** I did not trust the healthcare workers  **□** I did not want to pay for the vaccine  **□** There is risk of infection from unclean needles  **□** My dogs have already received a vaccine  **□** My dog is too young  **□** There is a risk of dog fight at the campaign  **□** It is unnecessary to spend extra time on dogs  **□** Other: _______________________________ | | |
| ***If the mop-up campaign was a door-to-door campaign:*** | | | | | | |
| 712 | | Did someone from the campaign come to your door? | | **□** Yes  **□** No 🡪 skip to 716  **□** I don’t know 🡪 skip to 716 | | |
| 713 | | Did you know what day the campaign was going to come to your door? | | **□** Yes **□** No | | |
| 714 | | Were any of your dogs vaccinated when the campaign came to your door? | | **□** Yes 🡪 skip to 716  **□** No  **□** I don’t know 🡪 skip to 716 | | |
| 715 | | *If no to 714,* Why weren’t any of your dogs vaccinated when the campaign came to your door?  *Don’t read. Assessor should listen to the respondent and then mark all that apply* | | **□** I did not know the campaign was coming  **□** It is not important to vaccinate against rabies  **□** I was unavailable or was not home when they came to my door  **□** I could not find my dogs  **□** My dogs already received the vaccine  **□** I do not trust the vaccine  **□** My dog is too young  **□** I did not want to pay for the vaccine  **□** It is unnecessary to spend extra time on dogs  **□** Other: _______________________________ | | |

716. Click "Link Form" below to add one form for each of your owned dogs in the household or dogs that are not yours, but you provide care for:

| **Dog** | **a. Age** | **b. Sex**  **-M**  **-F** | **c. Owned status:**  **1. Owned**  **2. Unowned** | **d. Confinement status:**   1. **Always on property** 2. **Roaming, sometimes** 3. **Roaming, always** | **e. Has this dog ever received a rabies vaccine?** | **f. Was this dog vaccinated against rabies in the past year?** | **g. For the most recent rabies vaccine this dog received, where did you get the vaccine from?**   1. **At vet facility** 2. **Vaccination campaign** 3. **Other, describe** | **h. Was this dog vaccinated in the recent campaigns?** |
| --- | --- | --- | --- | --- | --- | --- | --- | --- |
| 1. |  |  |  |  | ○ Yes  ○ No  ○ Don’t know | ○ Yes  ○ No  ○ Don’t know |  | ○ Yes  ○ No  ○ Don’t know |
| 2. |  |  |  |  | ○ Yes  ○ No  ○ Don’t know | ○ Yes  ○ No  ○ Don’t know |  | ○ Yes  ○ No  ○ Don’t know |
| 3. |  |  |  |  | ○ Yes  ○ No  ○ Don’t know | ○ Yes  ○ No  ○ Don’t know |  | ○ Yes  ○ No  ○ Don’t know |
| 4. |  |  |  |  | ○ Yes  ○ No  ○ Don’t know | ○ Yes  ○ No  ○ Don’t know |  | ○ Yes  ○ No  ○ Don’t know |
| 5. |  |  |  |  | ○ Yes  ○ No  ○ Don’t know | ○ Yes  ○ No  ○ Don’t know |  | ○ Yes  ○ No  ○ Don’t know |
| 6. |  |  |  |  | ○ Yes  ○ No  ○ Don’t know | ○ Yes  ○ No  ○ Don’t know |  | ○ Yes  ○ No  ○ Don’t know |

| End of survey. Thank you for your time and participation!!! |
| --- |
| 801. Please click here to enter any other comments or relevant information from the respondent or problems encountered during the survey itself. |
